# Gene Expression Patterns Unveil New Insights in Papillary Thyroid Cancer

**DOI:** 10.3390/medicina55080500

**Published:** 2019-08-19

**Authors:** Mihai Saftencu, Cornelia Braicu, Roxana Cojocneanu, Mihail Buse, Alexandru Irimie, Doina Piciu, Ioana Berindan-Neagoe

**Affiliations:** 1 Faculty of Medicine, Iuliu Hatieganu University of Medicine and Pharmacy, 40015 Cluj-Napoca, Romania; 2 Research Center for Functional Genomics and Translational Medicine, Iuliu Hatieganu University of Medicine and Pharmacy, 23 Marinescu Street, 40015 Cluj-Napoca, Romania; 3 MEDFUTURE-Research Center for Advanced Medicine, Iuliu Hatieganu University of Medicine and Pharmacy, Cluj-Napoca, Romania, 23 Marinescu Street, 40015 Cluj-Napoca, Romania; 4 Department of Surgery, The Oncology Institute “Prof. Dr. Ion Chiricuta”, 40015 Cluj-Napoca, Romania; 5 Department of Surgical Oncology and Gynecological Oncology, Iuliu Hatieganu University of Medicine and Pharmacy, Cluj-Napoca, Romania, 40015 Cluj-Napoca, Romania; 6 Department of Nuclear Medicine and Endocrinology, The Oncology Institute “Prof. Dr. Ion Chiricuta”, 40015 Cluj-Napoca, Romania; 7 Department of Oncology, Iuliu Hatieganu University of Medicine and Pharmacy, 40015 Cluj-Napoca, Romania; 8 Department of Functional Genomics and Experimental Pathology, The Oncology Institute “Prof. Dr. Ion Chiricuta”, Republicii 34th street, 400015 Cluj-Napoca, Romania

**Keywords:** thyroid papillary cancer, Cancer Genome Atlas (TCGA), gene expression and biological adhesion signature

## Abstract

*Background and objectives:* Papillary thyroid carcinoma is the most frequent variety of all malignant endocrine tumors. It represents a heterogeneous malignancy with various clinical outcomes, emphasizing the need to identify powerful biomarkers with clinical relevance. *Materials and Methods:* Available gene expression data (level 3) for thyroid cancers were downloaded from the Cancer Genome Atlas (TCGA), followed by bioinformatic analyses performed on the data set. *Results:* Based on gene expression analysis, we were able to identify common and specific gene signatures for the three main types of papillary thyroid carcinoma (classical, follicular variant, and tall-cell). The survival rate was not significantly different among the main subtypes, but we were able to identify a biological adhesion signature with impact in patient prognostic. *Conclusions:* Taken together, the gene expression signature and particular adhesion signature, along with ITGA10 and MSLN in particular, could be used as a prognostic tool with important clinical relevance.

## 1. Introduction

Thyroid cancer is the most common endocrine malignancy, showing an increasing incidence during the last years [1]. Papillary thyroid carcinoma (PTC) has the highest prevalence among all thyroid malignancies, representing around 80% of all thyroid carcinomas [2]. There are three common subtypes of papillary thyroid cancer: classical (conventional), follicular, and tall-cell [2]. The tall-cell phenotype is the most aggressive subtype, showing an increased risk of metastatic dissemination to cervical lymph nodes [2,3].

Genetic and epigenetic alterations are the driving forces of thyroid cancer. There has been an exciting advance in comprehending its molecular pathogenesis during recent years [1,3,4]. In spite of this fact, there are still various issues related to the pathogenic mechanisms behind thyroid cancer that need to be solved, such as the identification of various genes, mutations, epigenetic alterations, and environmental factors implicated in its progress [5,6,7]. To manage the limitations related to molecular pathogenesis, many researchers have attempted to identify useful genetic tools [1,4,8]. Such tools for transcriptomic analysis are expected to provide novel information with clinical relevance, in order to improve the management and the outcome of this disease [9,10,11].

The Cancer Genome Atlas (TCGA) is a large database of molecular data for a wide range of cancer types, including genome and transcriptome evaluation, selected in a laborious and consistent manner [7,12,13]. These furnish remarkable possibilities for complex data analyses and for detecting trends of alteration of transcriptomic patterns. This kind of analysis will lead to novel insight into the management of thyroid malignancies.

The current study was focused on the evaluation of global gene expression pattern in papillary thyroid carcinoma tissue and its related main types (classical, follicular, and tall-cell) compared with adjacent normal tissue, in order to identify differentially expressed genes. Then, we integrated the altered gene expression signature in biological context, trying to identify the main pathways and biological processes impacting the development of new biomarkers of thyroid cancer.

## 2. Materials and Methods

### 2.1. Gene Expression Evaluation Based on TCGA Data

Papillary thyroid cancer RNA sequencing data (systematized on its main subtypes) as well as matched adjacent noncancerous control data was used to evaluate the global alteration in gene expression pattern. This data was downloaded from the TCGA module database of the UCSC (University of California Santa Cruz) Xena browser, in the form of data matrices containing log2, normalized expression data, together with clinical and demographic information of the patients. We included 357 patients with classical PTC, 102 with the follicular variant of PTC, and 36 with tall-cell PTC (Table 1). We used the GeneSpring Gx analysis software (provided by Agilent Technologies, Santa Clara, CA, USA) in order to evaluate both the global and the characteristic subtypical alterations in PTC. As a cut-off value, we used a fold change of ±2 and a *p*-value < 0.05, corrected using the Benjamini–Hochberg method to restrain the false discovery rate (FDR) resulted in multiple testing.

### 2.2. Pathway Enrichment and Biological Process Analysis, Gene Ontology Classification, and Network Visualization

Pathway enrichment analysis, biological process analysis and gene ontology classification were performed for genes with an altered expression level by using the online Panther tool (http://www.pantherdb.org) [14]. STRING (https://string-db.org) and miRNET (https://www.mirnet.ca/miRNet/faces/home.xhtml) were used to determine gene involvement in PTC pathogenesis and for the inclusion of altered genes in key regulatory network.

## 3. Results

### 3.1. Differential Gene Expression in Tumor Tissues versus Normal Tissues for PTC 

Global gene expression was evaluated in tumor tissues (*n* = 505) versus normal tissues (*n* = 59), considering as cut-off value the FC (fold change) of ±2 and corrected *p*-value ≤ 0.05. A total of 1120 upregulated genes and 1191 downregulated genes were identified.

**Figure 1:**
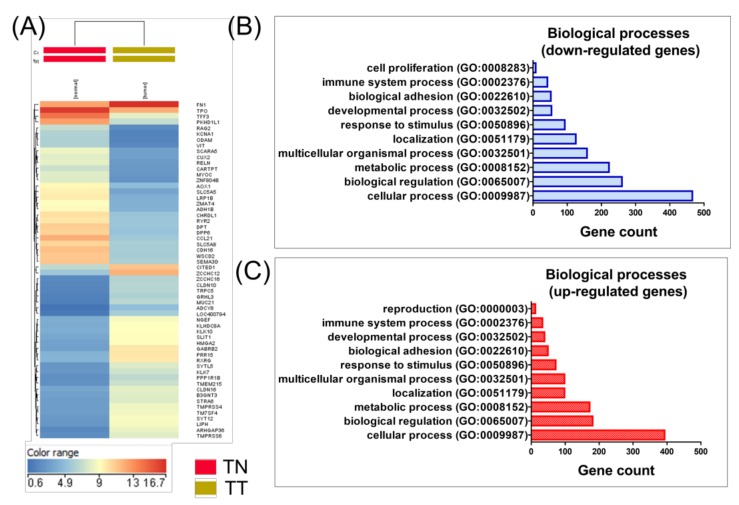
Hierarchical clustering and gene ontology classification for gene expression data in papillary thyroid cancer versus normal thyroid tissue. (**A**) Heatmap for papillary thyroid cancer, using malignant tissues (*n* = 505) and normal tissues (*n* = 59). Significantly overexpressed genes are highlighted in red and underexpressed genes are shown in blue, emphasizing distinct PTC subtypes with clinical and biological relevance; (**B**) and (**C**) Biological classification, based on the gene expression signature, in tumor versus normal tissue in PTC patients (by using the online PantherDB tool). (TN: normal thyroid tissue, TT-tumor thyroid tissue).

Figure 1A shows the hierarchical clustering of the genes on thyroid cancer, providing evidence that PTC can be classified on its own distinct expression pattern, emphasizing the diversity and heterogeneity of PTC. Significantly enriched biological processes of differentially expressed genes are presented in Figure 1B,C.

**Figure 2:**
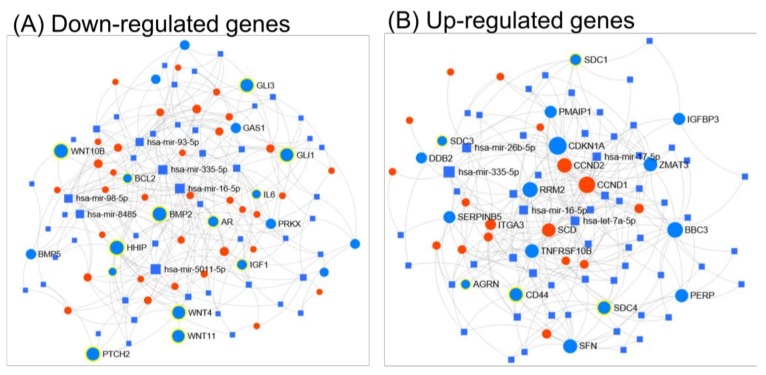
The representative miRNA-gene network interactions between the genes with an altered expression levels when comparing tumor to normal tissue and their respective miRNAs, generated using miRNet. (**A**) shows mRNA–miRNA network for down-regulated genes in PTC, involved in the Hedgehog signaling pathway (n = 15, *p*-value = 0.000212), Axon guidance (n = 18, *p*-value = 0.0359) and basal cell carcinoma (n = 10, *p*-value = 0.0373), while (**B**) shows mRNA–miRNA network for up-regulated genes in PTC, associated with the p53 signaling pathway (n = 16, *p*-value = 0.0000254), ECM-receptor interaction (n = 18, *p*-value = 0.0000254) and pathways in cancer (n = 29, *p*-value = 0.0935). The genes belonging to these aforementioned pathways are highlighted with blue dots, while the red dots represent the genes with altered expressions involved in other interconnected pathways. The blue squares represent the miRNAs that target the altered genes, where the size of the square is proportional to the number of signaling pathways involved (based on KEEG classification).

The functional pattern of miRNA–mRNA regulatory network has been shown to be involved in both tumor initiation and progression in several cancers, including PTC. Using a miRNET tool allowed us to emphasize the crucial role of the interconnection between the miRNA and mRNA networks, as well as taking into consideration the gene ontology classification (KEGG or Reactome). Based on KEGG (Kyoto Encyclopedia of Genes and Genomes) classification, most of the downregulated genes belong to the Hedgehog signaling pathway (*p*-value 0.000212), Axon guidance (*p*-value 0.0359), basal cell carcinoma (*p*-value 0.0373), displayed in Figure 2A. Reactome classification revealed the downregulation of genes related to thyroxine biosynthesis (*p*-value 0.00339), neuronal system (*p*-value 0.102) or developmental biology (*p*-value 0.102), presented in Figure S1A. Regarding up-regulated genes, KEGG classification reveals that up-regulated genes are involved in altered pathways belonging to the p53 signaling pathway (*p*-value 0.0000254), ECM (extracellular matrix)-receptor interaction (*p*-value 0.0000254) and pathways in cancer (*p*-value 0.0935), displayed in Figure 2B. Reactome classification of up-regulated genes reveals their involvement in extracellular matrix organization (*p*-value 1.74 × 10^−12^), degradation of the extracellular matrix (*p*-value 2.6 × 10^−10^) and collagen degradation (0.0000149), presented in Figure S1B.

### 3.2. Analysis of Gene Expression Pattern in the Main Types of Thyroid Cancer

**Figure 3:**
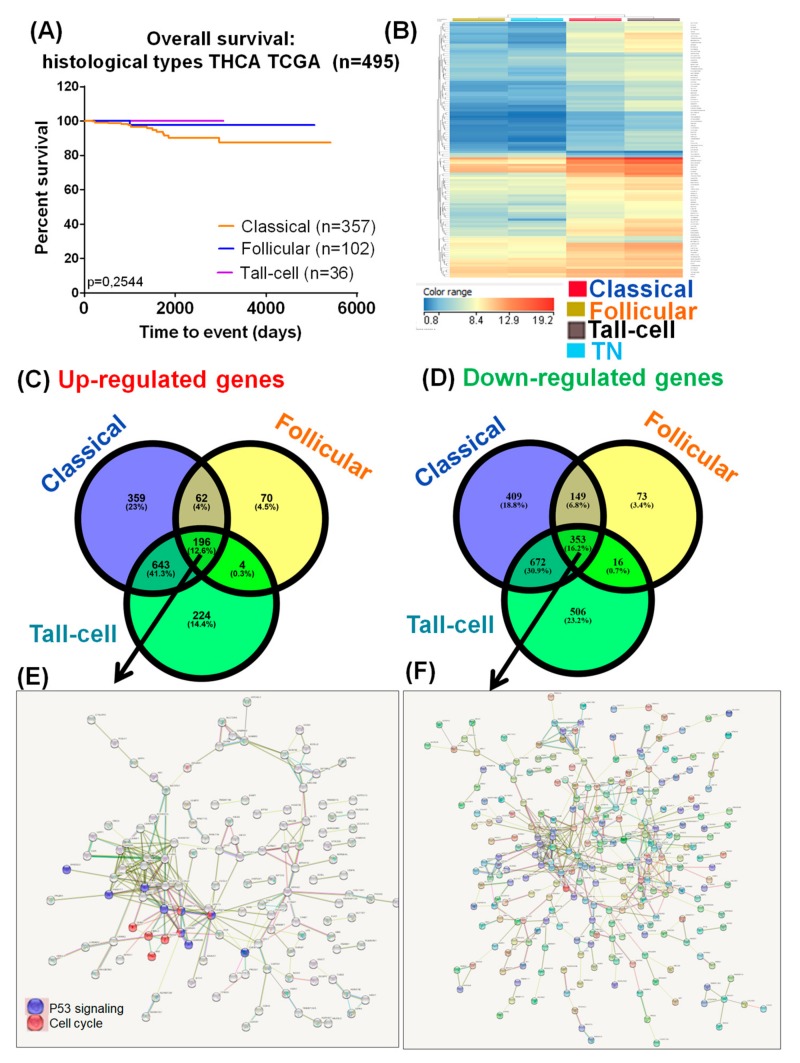
Molecular portrait of thyroid cancer. (**A**) Kaplan–Meier survival analysis, categorized by the main 3 types of papillary thyroid cancer; (**B**) Heatmap for specific altered genes for the three selected groups versus TN (normal thyroid tissue), considering *p*-value 1 × 10^−30^; (**C**,**D**) Venn diagram for identifying common and specific upregulated or downregulated genes in each subtype of PTC; (**E**) String network for the common 196 overexpressed genes; (**F**) String network for the common 353 downregulated genes.

The survival rates of patients with PTC were not significantly different (Figure 3A), despite the fact that important alterations among the three selected subtypes of PTC were observed (classical PTC versus adjacent normal tissue, follicular PTC versus adjacent normal tissue, and tall-cell PTC versus adjacent normal tissue). The frequencies of genes showing altered expression, according to the three main types of PTC, sorted by the upregulation and downregulation criteria, are graphically represented as a Venn diagram in Figure 3B. Among the three PTC we identified, 196 upregulated and 353 downregulated genes as common gene expression signature. Figure 3E,F emphasize the gene network for the common signature specific for the downregulated and overexpressed genes. These data will lead to the identification of new common players in PTC, emphasizing the important role of TP53 signaling and cell cycle regulators.

**Figure 4:**
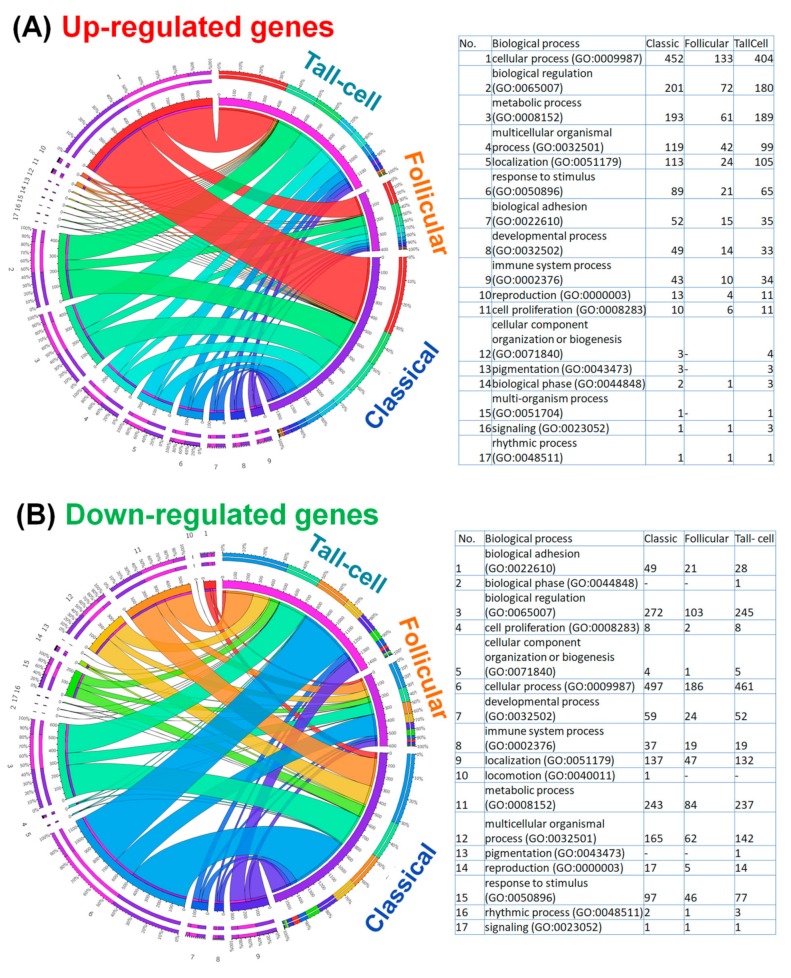
Circos plot, showing the association between the top (**A**) up-regulated and (**B**) down-regulated biological processes according to PTC subtypes. The main biological processes, and their specific gene ontology (GO) are listed as a table in the right panel of the figure, presenting the number of genes for each specific GO for the three PTC subtypes.

A GO enrichment analysis was performed (using the Panther online tool) in order to gain a better understanding of gene functions and signaling pathways in altered genes for the three main PTC subgroups. The top 10 enriched pathways of upregulated and downregulated genes (generated by the Panther gene ontology online tool) for the main three types of PTC are shown in Figure 4, this determining the basis of the main altered mechanisms that represent an important direction for future experimental research.

### 3.3. Analysis of Biological Adhesion Signature in Papillary Thyroid Cancer

Cellular adhesion mechanisms have an important role in PTC. These mechanisms are specifically activated in all the three subtypes of PTC, albeit more pronounced in the classical and tall-cell subgroups, while the follicular variant of PTC shows a less pronounced activation.

We performed pre-ranked gene set enrichment analysis (using the Panther online tool) for the three main subtypes of PTC in order to be able to functionally interpret the biological adhesion signatures. This gene list, annotated as ‘biological adhesion’, was integrated in STRING in order to determine the gene interactions among each specific group.

**Figure 5:**
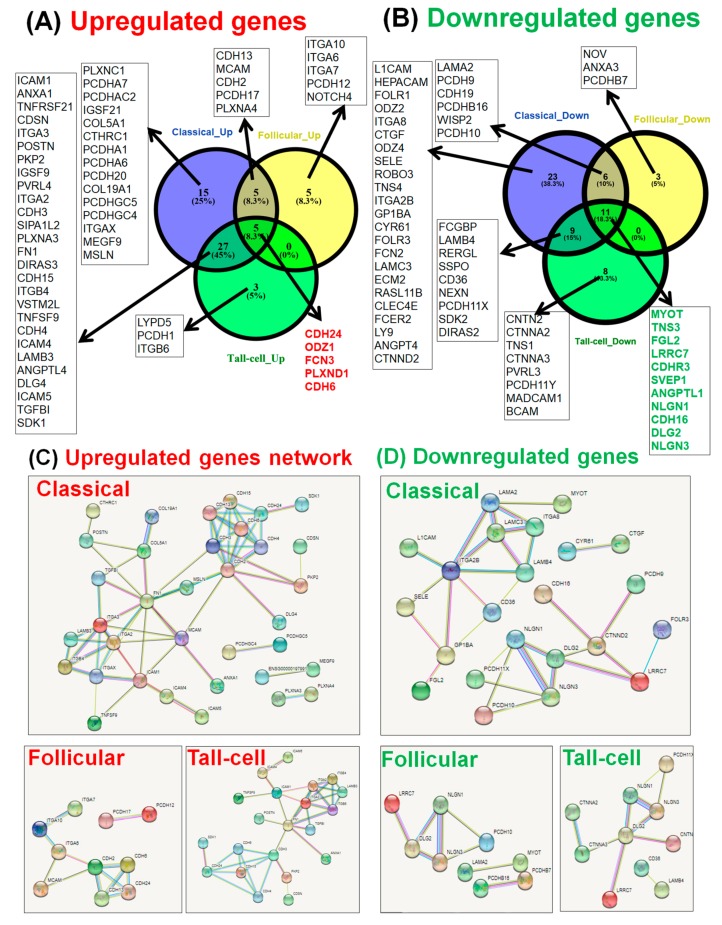
The involvement of biological adhesion molecules in papillary thyroid cancer. Venn diagram for identifying the specific and common (**A**) upregulated genes and (**B**) downregulated genes related to biological adhesion in classic, follicular variant, and tall-cell PTC. Biological adhesion molecules interaction network (generated using STRING): (**C**) upregulated genes and (**D**) downregulated genes.

This analysis is graphically represented by: a Circos plot for the overexpressed genes (Figure 4A) and downregulated genes (Figure 4B); a Venn diagram for the altered genes involved in biological adhesion (Figure 5A,B); and by an interconnected network (generated using the STRING tool) for the overexpressed and downregulated genes involved in biological adhesion (Figure 5C,D).

### 3.4. Association of Key Genes Expression Related to Biological with Survival of Patients with Papillary Thyroid Carcinoma

**Figure 6:**
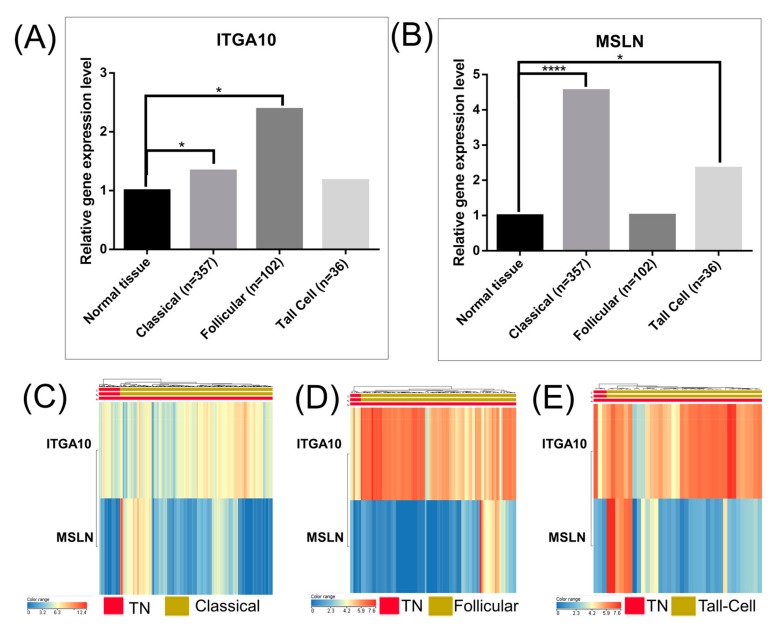
Bar graphs of the global expression levels of (**A**) ITGA10 (Integrin Subunit Alpha 10) and (**B**) MSLN (Mesothelin) expressions in the investigated PTC subtypes, generated using the TCGA data analysis (* *p* ≤ 0.05, ** *p* ≤ 0.01, *** *p* ≤ 0.001, **** *p* ≤ 0.0001). The heatmap representations of the individual expression levels of ITGA10 and MSLN in the investigated subtypes of papillary thyroid cancer: (**C**) classical, (**D**) follicular and (**E**) tall-cell versus TN (normal tissue).

Survival analysis based on TCGA data showed that the expression levels of CDH13, CDH24, CDH6, ICAM1, ITGA10, ITGA6, ITGA7, ITGAX, ITGB6, MCAM, MSLN, NOTCH4, and TGFBI genes were not associated with the overall survival rate of patients with follicular type and tall-cell PTC (Figure 6).

For the case of the ITGA10 gene, the FC was: 1.33 for the classical subtype (below the threshold limit cut-off of the FC ± 2, *p*-value = 0.021); 2.38 for follicular subtype (*p*-value = 0.025); and 1.174 for the tall-cell subtype (*p*-value = 0.874). Similarly, for the MSLN gene, the FC was: 4.54 for the classical subtype (*p*-value = 2.4 × 10^−8^); 1.01 for follicular subtype (*p*-value = 0.93); and 2.34 for the tall-cell subtype (*p*-value = 0.511). These results can be seen in Figure 6A,B, respectively.

**Figure 7:**
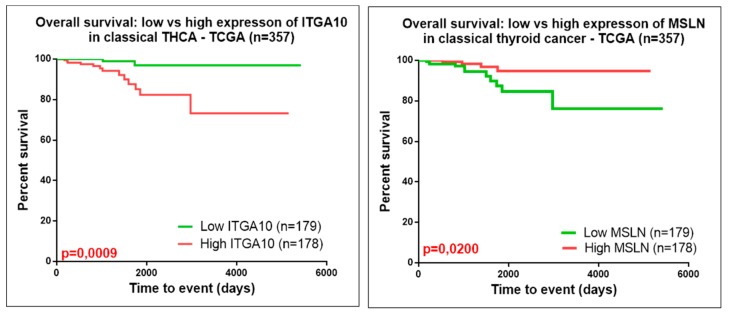
Kaplan–Meier survival analysis for high and low expression levels of ITGA10 and MSLN in classic PTC, based on TCGA data analysis.

The heatmap representations, seen in Figure 6C–E, emphasize the expression level in normal and tumor tissues for ITGA10 and MSLN. As can been seen in Figure 7, either the high gene expression of ITGA10 or low gene expression of MSLN was correlated to a significant decrease in the disease-free overall survival of patients with classical PTC. The rest of the evaluated genes presented no statistically significant correlation between overall survival rate and gene expression levels.

## 4. Discussions

In the present study, we evaluated and analyzed the difference of the expression profiles of mRNAs in PTC, emphasizing the common and specific miRNA signatures for its three main subtypes: classical, follicular, and tall-cell. These findings were also verified through functional and pathway enrichment analyses. Comparing the gene–gene and gene–miRNA interactions of the genes exhibiting an altered expression level is a useful method for both discerning dysregulated pathways in the process of the disease [15] and pointing out the crosstalk between signaling pathways, as can be seen in Figure 2. Expanding evidence shows that p53 family members lead to the advancement of thyroid cancer [16], and our research also identified activated genes related to this signaling pathway.

The malignant development and progression are influenced by the fact that thyroid hormone synthesis system is downregulated in PTCs due to de-differentiation [17], revealing the relation to thyroxine biosynthesis (Reactome classification). Recent studies have further shown promising outcomes following chemical reduction of thyroid hormones or inhibition, or their binding to the integrin receptor, to have positive impact in patient prognostic [17].

A previous study accentuated the importance of metabolic gene signatures in PTC [18]. Another similar study identified 719 genes with altered expression levels and, through KEGG pathway enrichment analyses of the overexpressed genes, demonstrated the association to focal adhesions, ECM-receptor interactions, adherents junctions, and 12 other pathways; all these overexpressed genes belong to biological adhesions mechanisms [19], and all these belong to biological adhesions mechanisms. These are directly related to key cellular processes including motility, proliferation, differentiation, regulation of gene expression, and cell survival [19,20], which are explicitly altered in many cancer types. Also, it has been previously stated that thyroid cancers can activate the immunologic pathway and biological adhesion pathways, a fact consistent with our observation in all three subtypes of PTC [21]. Cell adhesion molecules mediate important cellular interactions involved in tumor progression [22]. It was observed that the alteration in the gene expression pattern of cell adhesion molecules has been involved in all steps of tumor progression, including tumor cell detachment from primary site, cellular intravasation and extravasation [22,23].

In the case of downregulated genes, we observed a connection to the Hedgehog pathway, this emphasizing the dual role of these signaling pathways. As previously presented, it has the capacity to crosstalk with RAS/BRAF/MEK pathway and ligand secretion by tumor stroma, inducing cancer cell migration and in vitro tumorigenesis [24].

The classical PTC is a particular subtype where the alteration of adhesion molecules seems to have an important role in tumor progression. A previous profiling study revealed similar altered mechanisms, where overexpressed genes were related mainly to cell adhesion processes, protease binding, or ECM-receptor interactions [25]. ICAM-1 (Intercellular adhesion molecule 1) is an important molecule that has an important role in cell adhesion regulation and in the inflammatory response progression [26]. It was previously proven that ICAM-1 is upregulated in both classical and tall-cell PTC, therefore it could be considered a biomarker of PTC progression [26].

Although the previously established biomarkers for predicting clinical outcomes based on molecular markers related to adhesion molecules have not been a proof of success in clinical management of PTC, it is still relevant to determine the accurate and generalized predictive signatures in this type of cancer. Our study accentuated for the first time a correlation of the overall survival rate to ITGA10 and MSLN in classic PTC. ITGA10 was proved to have an important role in adhesion, migration and/or in the regulation of inflammatory responses [27]. MSLN was observed in several cancer types, meaning it represents an important therapeutic immunological target [28]. Inhibition of MSLN reversed mesenchymal features and attenuated stem cell properties, in addition to inhibiting tumor growth and metastasis in lung cancer models; this could be exploited also for thyroid cancer [29]. Therefore, additional biological adhesion and immunological genes could be utilized to estimate the prognostic impact of the different subtypes of thyroid cancer.

## 5. Conclusions

In conclusion, our work demonstrated that the transcriptomic evaluation and pathway analysis have an important role in understanding the mechanism related to tumorigenesis and tumor progression in papillary thyroid cancer. The specific and common gene expression signature provided better insights into the molecular characteristics of these malignancies.

This work demonstrated a methodology of database analysis for determining gene expression patterns useful for the identification of PTC patients specific subtypes based on biological adhesion signature (particularly TGA10 and MSLN). Further characterizing this signature could facilitate the discovery of novel prognostic and predictive factors that could guide a personalized treatment approach to PTC. Forthcoming investigations should attempt to clinically and experimentally validate mRNA expression-based adhesion molecules’ expression level by PTC subtypes. We are looking forward to additionally clarifying their biological relevance through further validation.

## Figures and Tables

**Table 1:** Demographic, pathologic and clinical characteristics of TCGA samples for PTC.

Demographics		Papillary Thyroid Carcinoma (*n* = 505)							
		Classical (*n* = 358)		Follicular (*n* = 102)		Tall Cell (*n* = 36)		Other (*n* = 9)	
		Nr	%	Nr	%	Nr	%	Nr	%
Sex	Males	98	27.37	24	23.53	9	25.00	5	55.56
	Females	260	72.63	78	76.47	27	75.00	4	44.44
Age	Median, Range	45.5, 15–88		46, 16–83		46, 28–89		46, 22–62	
	Median, Range ♂	51, 15–88		51, 16–80		51.5, 38–85		37, 22–60	
	Median, Range ♀	43.5, 15–87		46, 17–83		46, 28–89		46, 42–62	
Focus type	Unifocal	203	56.70	51	50.00	11	30,56	3	33.33
	Multifocal	148	41.34	50	49.02	23	63.89	6	66.67
	Unknown	7	1.96	1	0.98	2	5.56	-	
TNM	T1	103	28.77	32	31.37	7	19.44	1	11.11
	T2	122	34.08	40	39.22	3	8.33	1	11.11
	T3	111	31.01	28	27.45	25	69.44	7	77.78
	T4	20	5.59	2	1.96	1	2.78	-	
	Tx	2	0.56	-		-		-	
	T unknown	-		-		-		-	
	N0	149	41.62	65	63.73	13	36.11	3	33.33
	N1	184	51.40	13	12.75	22	61.11	6	66.67
	N2	-		-		-		-	
	Nx	25	6.98	24	23.53	1	2.78	-	
	N unknown	-		-		-		-	
	M0	222	62.01	32	31.37	23	63.89	5	55.56
	M1	4	1.12	5	4.90	-		-	
	Mx	131	36.59	65	63.73	13	36.11	4	44.44
	M unknown	1	0.28	-		-		-	
Tumor stage	I	212	59.22	56	54.90	10	27.78	6	66.67
	II	29	8.10	22	21.57	-		1	11.11
	III	73	20.39	18	17.65	20	55.56	1	11.11
	IV	43	12.01	5	4.90	6	16.67	1	11.11
	Unknown	1	0.28	1	0.98	-		-	
Anatomic site	Right lobe	141	39.39	59	57.84	12	33.33	3	33.33
	Left lobe	132	36.87	30	29.41	12	33.33	2	22.22
	Bilateral	63	17.60	10	9.80	9	25.00	4	44.44
	Isthmus	17	4.75	2	1.96	3	8.33	-	
	Unknown	5	1.40	1	0.98	-		-	
	Goiter	4	1.12	-		-		-	
	Graves’ disease	1	0.28	-		1	2.78	-	
	Hashimoto Thyroiditis	1	0.28	-		-		-	
	Hashimoto Thyroiditis & Hypothyroidism	-		1	0.98	-		-	
	Hyperthyroidism	3	0.84	-		-		-	
	Hypothyroidism	5	1.40	3	2.94	2	5.56	1	11.11
	Lymphocytic Thyroiditis	32	8.94	11	10.78	-		-	
	Lymphoid hyperplasia	-		1	0.98	-		-	
	Nodular Hyperplasia	47	13.13	35	34.31	7	19.44	2	22.22
	Other	8	2.2	2	1.96	-		-	
	Normal	209	58.38	43	42.16	22	61.11	6	66.67
	Unknown	48	13.41	6	5.88	4	11.11	-	
Response to therapy	Complete response	107	29.89	31	30.39	20	55.56	5	55.56
	Partial response	15	4.19	3	2.94	-		-	
	Radiological progressive disease	5	1.40	-		1	2.78	-	
	Stable disease	2	0.56	-		2	5.56	-	
	Unknown	229	63.97	68	66.67	13	36.11	4	44.44

TNM-tumour stage, lymph nodes, metastases.

## Supplementary Materials

The following are available online at https://www.mdpi.com/1010-660X/55/8/500/s1, Figure S1. Interconnected gene network with specific miRNAs, generated using miRnet. (A) mRNA–miRNA network for down-regulated genes in PTC (based on Reactome classification) was related to thyroxine biosynthesis, neuronal system or developmental biology; (B) up-regulated genes in PTC (based on Reactome classification) were related to extracellular matrix organization, degradation of the extracellular matrix, and collagen degradation. Figure S2. Bar graphs, displaying the enriched GO of differential expressed genes (using the Panther Gene Ontology online tool) for the main subtypes of papillary thyroid cancer: (A) classic, (B) follicular variant and (C) tall-cell.
