# Supplementary material for: Gene Expression Patterns Unveil New Insights in Papillary Thyroid Cancer
Source: Medicina (Kaunas). 2019 Aug 19;55(8):500. doi: 10.3390/medicina55080500 (PMC6723230; doi:10.3390/medicina55080500)
Supplement: Supplementary file 1 [file medicina-55-00500-s001.pdf]

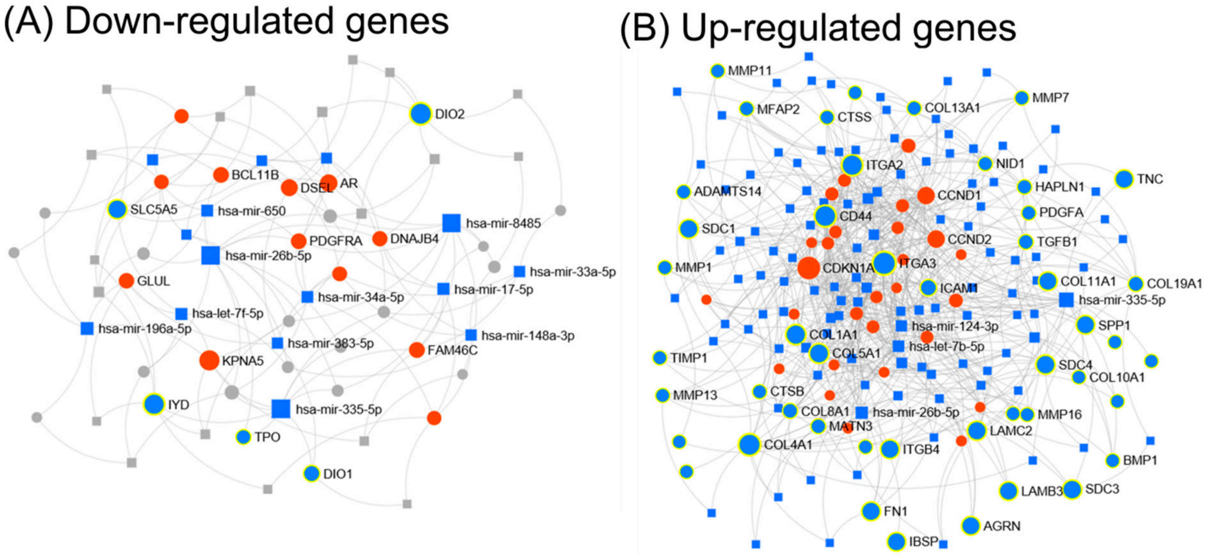

Figure S1. Interconnected gene network with specific miRNAs, generated using miRnet. (A) mRNA-miRNA network for down-regulated genes in PTC (based on Reactome classification) was related to Thyroxine biosynthesis, Neuronal System or Developmental Biology; (**B**) up-regulated genes in PTC (based on Reactome classification) were related to extracellular matrix organization, degradation of the extracellular matrix, collagen degradation.

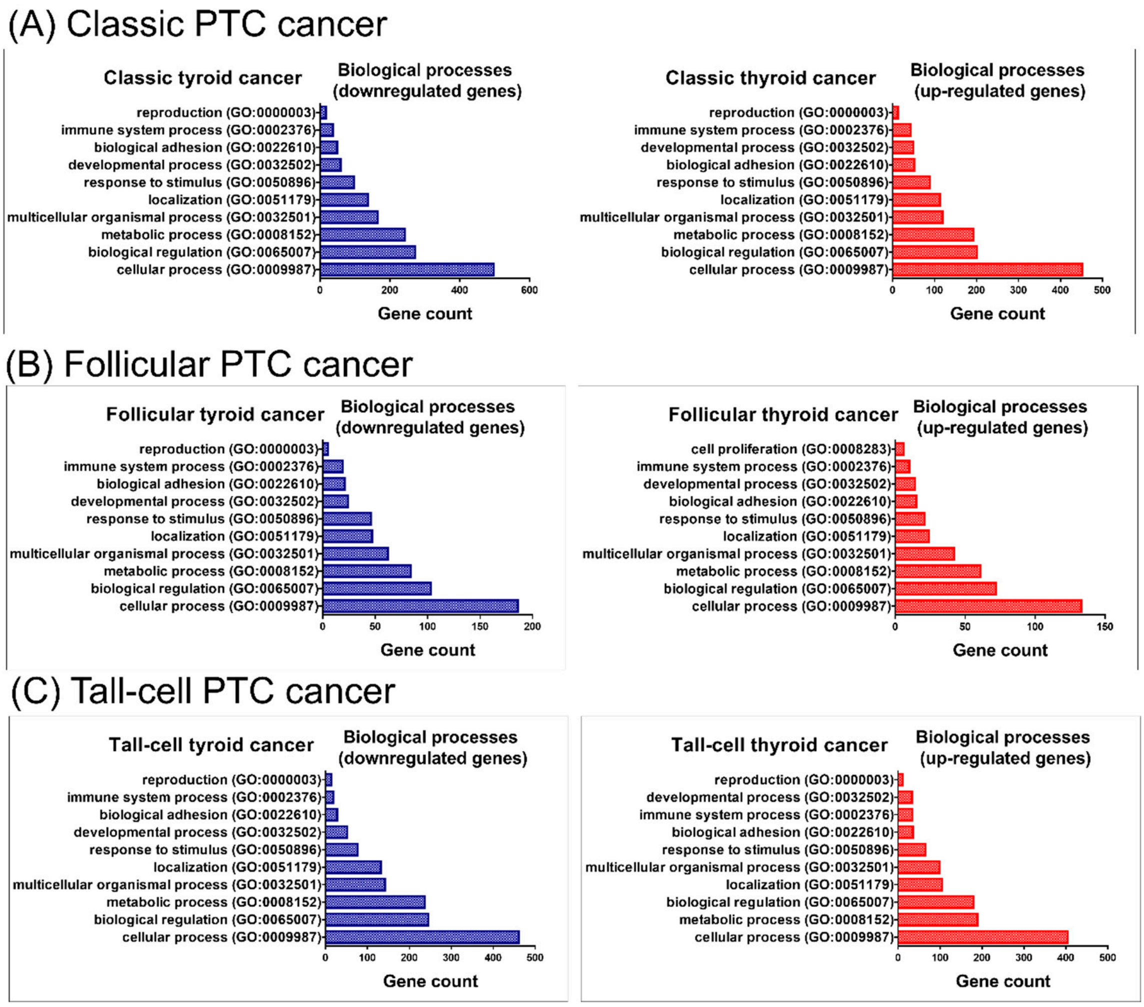

Figure S2. Bar graphs, displaying the enriched GO of differential expressed genes (using Panther Gene Ontology online tool) for the main subtypes of papillary thyroid cancer: (A) classic, (B) follicular variant and (C) tall-cell.
